# Kinematic Biomarkers of Functional Disability in Older Adults: Analysis of the Timed Up and Go Test

**DOI:** 10.3390/bios15090621

**Published:** 2025-09-19

**Authors:** Juliana Moreira, Bruno Cunha, José Félix, Rubim Santos, Andreia S. P. Sousa

**Affiliations:** 1 CIR, E2S, Polytechnic of Porto, Rua Dr. António Bernardino de Almeida, 4200-072 Porto, Portugal; jmo@ess.ipp.pt (J.M.); josefelixfelix15@gmail.com (J.F.); rss@ess.ipp.pt (R.S.); 2 Research Center in Physical Activity, Health and Leisure, Faculty of Sports, University of Porto, 4200-450 Porto, Portugal; 3 CINTESIS@RISE, CINTESIS.UPT, Portucalense University, Rua Dr. António Bernardino de Almeida 541, 4200-072 Porto, Portugal; bruno.cunha@upt.pt; 4 Department of Physiotherapy, Institute of Health of the North—Escola Superior de Saúde do Vale do Ave (ESSVA), Cooperativa de Ensino Superior Politécnico e Universitário (CESPU), 4760-409 Vila Nova de Famalicão, Portugal; 5 Department of Medical Sciences, University of Aveiro, Agras do Crasto, Campus Universitário de Santiago, 3810-193 Aveiro, Portugal

**Keywords:** functional test, kinematic biomarkers, factor analysis, aging, biomechanics

## Abstract

The Timed Up and Go (TUG) test is used to assess mobility in older adults, but its reliance on completion time limits its insight into detailed movement patterns that could serve as early indicators of functional decline. This study aimed to identify lower limb and trunk kinematic biomarkers during the TUG test that distinguish between older adults with and without functional disability, emphasizing the potential for wearable sensor applications. Sixty adults aged 60+ participated in this cross-sectional study. Three-dimensional lower limb and trunk range of motion (ROM), velocity, center of mass (CoM) displacement, and velocity were analyzed using an optoelectronic system across TUG subphases: sit-to-walk, walk-forward, turn, walk-back, and turn-to-sit. Principal component analysis identified eleven principal components (PCs), explaining 84.33% of the total variance. PCs included sagittal hip and knee motion and CoM velocity during turn-to-sit and walking (PC1); tri-dimensional trunk velocity during turning, walk-back, and sit-to-walk transitions (PC2, PC4, PC6); sagittal knee and hip velocity in sit-to-walk (PC3); and frontal and transverse plane knee ROM and velocity during turning (PC5). Significant differences between functional disability groups were found for PC1 and PC4. These findings provide benchmark data for developing and validating wearable biosensors aimed at monitoring kinematic biomarkers.

## 1. Introduction

Advances in wearable devices, including miniaturization, materials, and wireless communication, enable the collection of large-scale data that can enhance and transform our understanding of population health patterns [1,2,3]. These devices, capable of continuous monitoring and embedded diagnostics, are valuable for detecting early functional decline and enhancing functional assessments such as the instrumented Timed Up and Go (iTUG) test across clinical, community, and home settings [3,4,5].

The classical Timed Up and Go (TuG) test, developed by Podsiadlo and Richardson in 1991, is as a simple yet effective measure of dynamic balance and functional mobility that records [5] the time required to stand up from a standard chair, walk three meters, turn, return, and sit down [6]. Over the past two decades, the TuG test has been widely applied in both clinical and home settings [7] as a reliable measure of functional mobility in older adults [8], with established cut-off values predictive of future disabilities in both the basic activities of daily living (ADLs) and the instrumental activities of daily living (IADLs) [9,10]. However, research on kinematic performance in adults aged 60 or more has largely compared them to younger adults [11,12] or to individuals with Parkinson’s disease [5], rather than directly contrasting those with and without functional limitations.

Compared to younger adults, older adults require significantly more time to complete the turning and sitting subtasks, exhibit higher trajectories, reduced body inclination during the turning [11], and decreased ankle plantarflexion and hip flexion range of motion (ROM) [12]. Studies examining performance in specific phases of the TuG test [13,14,15,16,17] further show that rather than employing a pivot shift strategy, older adults move more slowly, require more steps, take longer to complete tasks, exhibit shorter step length and prolonged step duration [13,14], and demonstrate smaller spatial parameters, an extended double-support phase, and a higher stance-to-cycle ratio during turning gait maneuvers [15]. The sit-to-walk task has also been extensively studied, with a systematic review identifying three distinct strategies: forward continuation, balance, and sit-to-stand-to-walk [16]. In contrast, fewer studies have examined walk and turn-to-sit kinematics, highlighting that older adults exhibit reduced displacement angles and velocities in the lumbar spine, head, and knee, as well as earlier onset of lumbar movement and lateral head flexion during turning compared to younger adults [17].

Given the strong evidence supporting the predictive value of the TuG test for disability [9,10], it is essential to identify the parameters influencing performance, particularly by directly comparing older adults with and without functional disability. However, few studies have conducted such comparisons, and only a limited number have employed advanced technological systems [5]. A systematic review identified only two studies utilizing optoelectronic systems, one in patients before and after total hip arthroplasty [18] and another in Parkinson’s disease [19], highlighting the need to determine which biomarkers should be captured by wearable sensors, as most research focuses solely on fall risk [20,21]. Incorporating validated mobility metrics into wearable devices could enable continuous, remote, and personalized monitoring [4,5]. Additionally, research employing gold-standard technology to TuG performance in older adults remains scarce, with most studies comparing them to younger adults or individuals with Parkinson’s disease. This study therefore aims to characterize the kinematic performance of older adults and to differentiate the profiles of those with and without functional disability. To guide the identification of kinematic biomarkers for future wearable sensor applications, we analyzed the three-dimensional ROM of the lower limb joints and the trunk, the angular velocity, and the displacement and velocity of the center of mass (CoM) across the sit-to-walk, walk-forward, turn, walk-back, and turn-to-sit subphases of the TUG test. Principal component analysis (PCA) was used to identify variables with significant variance (loading > 0.8), which were then integrated into a principal component model (PCM) and compared between groups.

## 2. Materials and Methods

### 2.1. Study Design and Participants

This cross-sectional study adhered to the Strengthening the Reporting of Observational Studies in Epidemiology (STROBE) guidelines [22], and its protocol was registered in the ClinicalTrials.gov database (registration identifier: NCT05611723). This study was conducted at the Center for Rehabilitation Research, School of Health, Polytechnic of Porto.

A sample selection questionnaire was distributed across partner institutions of the research center between 1 June 2022 and 31 March 2023. Older adults who met all the eligibility criteria and voluntarily agreed to participate were contacted. Data collection was carried out in the movement laboratory of the research center from 1 June 2022 to 30 April 2023.

The inclusion criteria were as follows: aged 60 years or over; community-dwelling status; and the ability to perform gait, sit-to-stand, and stairs negotiation. Volunteers were excluded if they met any of the following criteria: institutionalization; established diagnosis of malignancy or terminal illness with an expected survival of less than one year; history of stroke, cerebral hemorrhage, head trauma, or Parkinson’s disease; cognitive impairment, defined as a score below 22 for 0-to-2 years of literacy, 24 for 3-to-6 years of literacy, and 27 for more than 6 years of literacy on the Mini Mental State Examination (MMSE) (Portuguese version) [23]; rheumatic conditions affecting the task performance; diabetic foot, lower limb fracture within previous six weeks, or other related conditions; unstable cardiovascular disease; hepatic or renal function failure; body mass index greater than 30 kg/m^2^; uncorrected vestibular and/or audition or vision impairments affecting task performance; or the presence of symptoms at the time of evaluation that could influence performance.

### 2.2. Sample Characterization

A questionnaire was administered to collect demographic information, health conditions, and fall history over the previous 12 months, along with self-reported medication use, and physical activity levels. Anthropometric data were obtained through bioimpedance analysis for body mass (Tanita Inner Scan BC-601; Tanita Europe B.V., Hoogoorddreef 56E; 1101 BE; Amsterdam; The Netherlands, precision 0.1 kg) and a stadiometer for height, (seca^®^ 222; seca—Medical Scales and Measuring Systems^®^, Birmingham, UK; accuracy 1 mm), from which the body mass index was calculated. Cognitive function was assessed using the MMSE, with scores ranging from 0 to 30.

Polypharmacy was defined as the use of five or more prescription medications [24]. Physical activity was evaluated with the validated Portuguese International Physical Activity Questionnaire: Short Form (IPAQ-SF) [25].

### 2.3. Disability Status Characterization and Assessment

Participants were divided into two groups: older adults without functional disability and those presenting impairments in two or more disability indicators of the previously identified in the literature [26]. Participants were classified as having functional disability if they met at least two of the following indicators: poor self-reported health, handgrip strength < 27 kg (men) or <16 kg (women), one-legged stance test < 10 s, and limitations on basic ADL (Barthel Index < 20) or in instrumental ADL (Lawton IADL < 23).

Handgrip strength was measured using the Jamar^®^ Plus+ Digital handheld dynamometer (Performance Health Supply, Cedarburg, WI, USA). Participants were seated, with the shoulder adducted, the elbow flexed at 90 degrees and unsupported, the forearm neutral, and the wrist extended at 30 degrees, following the guidelines of the American Society of Hand Therapists [27].

Balance was assessed using the one-leg stance test (OLST), where the total standing time on the participant’s preferred leg, with eyes open, was recorded in seconds. The test ended when participants could no longer maintain the position or reached the maximum duration of 60 s. Imbalance was noted when the lifted leg touched the ground or the participant lost balance [28].

Overall self-reported health status (SRH) was determined by the question, “In general, how do you rate your health today?” Responses were categorized as “very bad,” “bad,” “fair,” “good,” or “very good.” For analysis, SRH was dichotomized into “good” (including “very good” and “good”) and “poor” (including “fair,” “bad,” and “very bad”) [29].

The Barthel Index [30,31] was used to evaluate independence in basic ADLs, with scores ranging from 0 (complete dependence) to 20 (functional independence) [32].

To assess functional capacity in IADLs, the Lawton and Brody IADL Scale was used [33,34].

### 2.4. Kinematic Assessment

TuG test performance was recorded using the Qualisys Track Manager (Qualisys AB^®^, Göteborg, Sweden), with twelve optoelectronic cameras (eight Oquos500, four MiqusM3), one Miqus video camera (Qualisys AB^®^, Göteborg, Sweden), and two force plates (FP4060-08/10, Bertec^®^ Columbus, OH, USA). Kinematic data were collected at a 100 Hz frequency. Before testing, the researcher verbally explained the test procedure, and reflective markers were attached to the participants body according to a set up previously described in Moreira et al. [35]. Participants were instructed to stand up from a standard chair (seat height: 46 cm), walk at a comfortable pace to a line three meters away, turn around, walk back, and sit down, starting with their backs against the chair and without using their arms to rise. Each participants completed one familiarization trial prior to the test, followed by three valid recorded trials performance [6,36].

### 2.5. Data Processing and Kinematic Parameters

The marker trajectories were processed using Qualisys Track Manager software (Qualisys AB^®^, Sweden 2020.3). The processed data was then exported to Visual3D Professional™ (Has-Motion, Inc., Kingston, ON, Canada), which generated a full-body biomechanical model [35,37,38].

**Figure 1:**
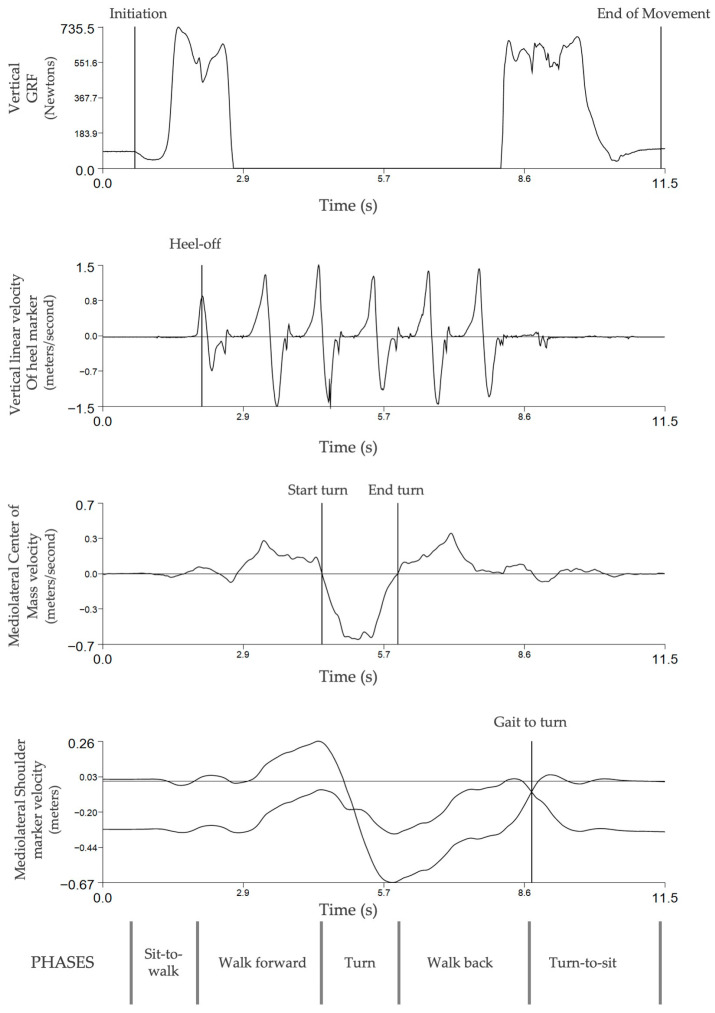
Timed Up and Go phases and respective events definitions according to the vertical ground reaction force (GRF), the vertical linear velocity of the heel marker, the mediolateral center of mass velocity, and the mediolateral shoulder marker velocity.

The TuG test was divided into five phases: sit-to-walk, walk forward, turn, walk back, and turn-to-sit [18,39] (Figure 1). The sit-to-walk initiation instance (T0—Initiation) was defined as the instant of the first change in the vertical ground reaction force (value equal to or lower than the mean minus 2 standard deviations of the baseline value computed for each individual) [40]. The end of this transition (T1—Heel-off) was defined as the moment when the heel of the swing limb loses contact with floor, corresponding to the maximum value of the anteroposterior linear velocity of the heel marker [41]. The walk-forward phase initiated at T1 (i.e., Heel-off) and ended when the CoM mediolateral velocity crossed the zero value, inverting its signal (T2—Start turn). The end (T3—End turn) of the turn phase were defined as the instant when the CoM velocity inverted the signal for a second time and the participant reached a distance of 0.7 m from the target [11]. The walk-back phase started at the end of turn (T3—End turn) and ended when the left and right shoulder marker mediolateral velocity equalized (T4—Gait to turn). The turn-to-sit phase started at T4 and ended before sitting, i.e., at the first deflection of vertical ground reaction force (GRF) (value equal to or lower than the mean minus 2 standard deviations of the baseline: T5—End of movement), as it is difficult to distinguish between the conclusion of the turn and the beginning of the sitting phase [18].

To characterize TuG performance, the values from task subphases and the completion time (seconds) were computed. For all phases of the TuG test, the trunk, hip, knee, and ankle ROM and the range of angular velocity were computed. These measures were obtained by subtracting the maximum value of each parameter to its minimum. The same computation was applied to the CoM position and velocity to calculate the CoM displacement and CoM velocity range. Lower limb and trunk joint angles were measured in degrees and calculated, as recommended by the ISB for the lower limb [42,43]. The joint angular velocities of trunk, hip, knee, and ankle movements were computed in degrees per second, describing the relative angular velocity of one segment relative to another segment [42]. The CoM was computed, including all body segments, considering the length of the body segments according to their joint centers, and the position was computed using the laboratory coordinate system.

### 2.6. Data Analysis

IBM SPSS Statistics for Mac OS, Version 29.0.2.0, was used to analyze the data, and a significance threshold of 0.05 was used for the analysis of statistics. The Shapiro–Wilk and Kolmogorov–Smirnov tests were used to assess the normality of the distribution of the disability indicators, the clinical characteristics, and the demographics for groups of older adults with and without disabilities, respectively. Independent sample *t*-tests or Mann–Whitney tests were used, where applicable, to evaluate between-group comparisons. To examine group relationships for categorical variables, specifically, sex, fall history, and SRH, chi-squared tests were utilized.

PCA was conducted on the tridimensional lower limb joint and trunk ROM, the angular velocity ranges, and the CoM displacement and velocity ranges to construct distinct PCMs for each phase of the TuG test. Potential outliers were manually inspected, and two individuals were identified as extreme outliers across several parameters in different TuG phases. One individual exceeded the interquartile range above the third quartile three times across seven parameters during the sit-to-walk phase and one parameter during the turn phase. The other individual was an outlier in three parameters during sit-to-walk, one during walk-forward, and four during walk-back. Both were therefore excluded from PCMs.

To ensure that each variable contributed equally to the analysis, a correlation matrix was employed. The dataset’s suitability for PCA was evaluated using the Kaiser–Meyer–Olkin (KMO) measure of sampling adequacy and Bartlett’s test of sphericity, which assesses the data’s appropriateness for dimensionality reduction. PCA was considered appropriate if Bartlett’s test produced a statistically significant result (*p* < 0.05) and KMO values exceeded 0.5.

Principal components (PCs) were extracted for each PCM phase using eigenvalues greater than 1, capturing the majority of the variance. To enhance interpretability, a Varimax rotation was applied [44]. The rotated component matrix was then analyzed to identify clusters of variables loading onto the same components, with a focus on variables showing high loadings (loading > 0.8), as recommended by Hair et al. (1998) [45]. Finally, a TuG PCM was developed by aggregating the parameters with loadings exceeding 0.8 for each phase.

The Mann–Whitney U test was used to assess differences between older adults with and without disabilities across each of the key principal components of the TuG PCM. Effect sizes were expressed as Cohen’s d, calculated from group means and pooled standard deviations, to aid interpretation and comparability with other studies.

### 2.7. Ethical Considerations

All participants received detailed information about this study’s purpose and methodology and provided written informed consent in accordance with the principles of the Declaration of Helsinki [46]. This study was submitted to the Institutional Ethics Committee on 27 April 2022 and approved on 25 May 2022 (registration number: CE0064C). Confidentiality was ensured by assigning a unique code to each participant’s data, which was securely stored on a password-protected computer accessible only to the investigator.

## 3. Results

**Figure 2:**
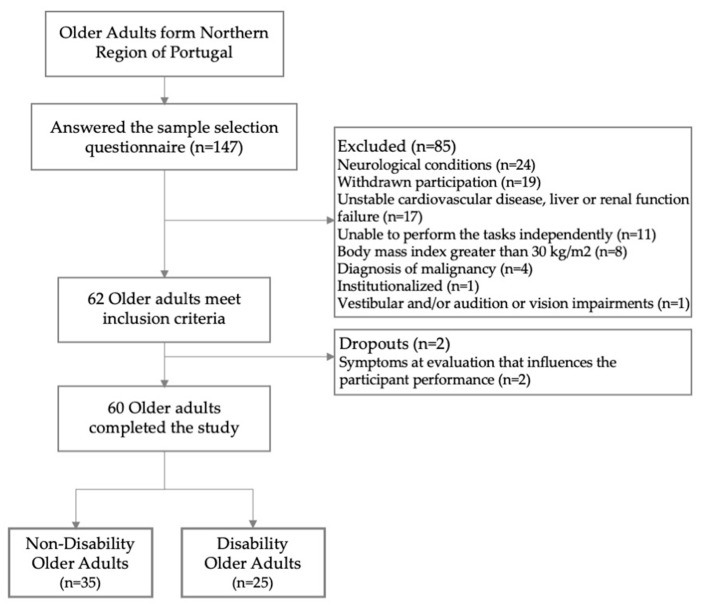
Flowchart illustrating the participant selection process.

This study initially enrolled 147 older adults, with 62 participants actively taking part, as illustrated in Figure 2. However, symptoms in two individuals impeded their ability to complete the required tasks during the examination. Consequently, the final analysis included data from 60 participants. Descriptive statistics summarizing their demographic and clinical characteristics are presented in Table 1.

The older adult participants had an average age of 67.86 years. Functional disability was associated with several differences, including reduced independence in ADL and IADL activities (*p* = 0.013 and *p* = 0.002, respectively), decreased hand grip strength (*p* = 0.018), shorter one-leg stand durations (*p* < 0.001), and poorer self-rated health (*p* < 0.001). Other demographic and clinical characteristics showed no substantial differences between the two groups, except for polypharmacy.

The older adults took approximately 11 s to complete the TuG test, with older adults experiencing functional disability requiring significantly more time than their counterparts (Table 2). Each phase of the TuG test was also significantly prolonged in the functional disability group, except for the turn and turn-to-sit phases, which did not show a statistically significant difference.

**Figure 3:**
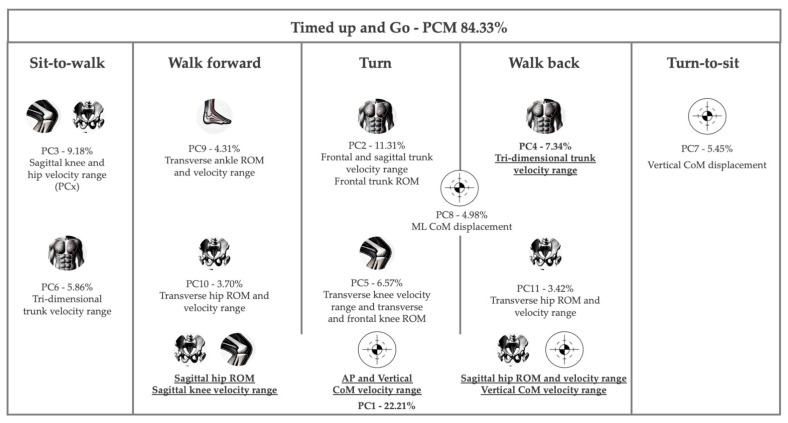
Principal component analysis descriptive diagram. Parameters with differences between older adults with and without functional disability are underlined and in bold.

Tables S1–S5 in the Supplementary Materials present the results of the preliminary PCMs, which assess the three-dimensional ROM and angular velocity of lower limb joints and the trunk, along with the displacement and velocity of the center of mass (CoM) across various TuG phases. The five PCMs produced KMO values ranging from 0.556 to 0.754, confirming this study sample’s suitability for dimensionality reduction. Additionally, Bartlett’s test of sphericity was significant (*p* < 0.001), reinforcing the appropriateness of factor analysis. The PCMs explained between 76.44% and 82.08% of the total data variance, with the walk-forward PCM accounting for the highest percentage. Across the different TuG phase PCMs, 59 variables exhibited factor loadings above 0.8. However, to maintain a KMO value above 0.5, 21 variables with communalities below 0.800—indicating a lower proportion of variance explained by the factors—were excluded, ensuring the retention of only variables with meaningful contributions to the extracted components. PC1 primarily included variables from hip, knee, and CoM velocity during the walk-back, walk-forward, and turn phases, explaining 22.21% of the total variance (Table 3 and Figure 3). PC2 encompassed tri-dimensional trunk velocity at the turn phase, as well as PC4 in the walk-back phase and PC6 in the sit-to-walk phase. PC3 included sagittal knee and hip velocity during sit-to-walk phase. PC5 included the transverse knee ROM and velocity range, along with frontal knee ROM, during the turn phase. PC7 included only vertical CoM displacement during the turn phase, while PC8 included mediolateral CoM displacement during the turn and walk-back phases. PC9 and 10 included variables from the walk-forward phase in the transverse plane, particularly ankle and hip ROM and velocity range, respectively. Additionally, transverse hip ROM and velocity range were also loaded onto PC11 but were associated with the walk-back phase.

The PC1 and PC4 scores differed between older adults with and without functional disability, according to the PC score analysis (Table 4 and Figure 3). The effect size analysis revealed that differences between groups were most evident in the first principal component (PC1), which showed a moderate effect (Cohen’s d = 0.59; 95% CI [0.06, 1.12]).

## 4. Discussion

This cross-sectional study aimed to characterize the kinematic performance of older adults during the TuG test and to differentiate the kinematic profiles of those with and without functional disability, with the broader goal of informing future wearable sensor applications for real-world monitoring. To achieve this, a comparative time analysis and PCA were conducted.

On average, older adults with functional disability required one additional second to complete the TuG test compared to those without disability. Both groups exceeded the 10 s threshold, a previously identified cut-off for functional dependency [47]. However, as multiple cut-off values have been proposed in the literature to predict functional disability [9,10], a more detailed analysis of the task-level analysis was warranted. Accordingly, each phase of the TuG test was timed, revealing that the functional disability group took significantly longer in the sit-to-walk, walk-forward, and walk-back phases. Although increases in time were also observed in the turn and turn-to-sit phases, these differences were not statistically significant. Previous studies have reported that older adults require an increased time ratio to complete turning and sitting subtasks compared to younger adults, often exhibiting extended turn trajectories [11]. It has also been documented that older adults employ a compensatory and conservative strategy, the sit-to-stand-and-walk, in which they reach an upright position and briefly pause before initiating gait [16]. The findings of the present study suggest that this stability-prioritizing strategy may be further exacerbated in older adults with functional disability. Additionally, prolonged time in the walk-forward and walk-back phases align with findings from studies on frail versus non-frail older adults, where reduced gait speed was associated with increased phase durations [39]. This slower gait speed is also characteristic of older adults with functional disability [48,49,50]. Various studies incorporate time-based metrics in wearable monitoring, primarily to predict fall risk [20,21]. Integrating the findings of the present study could enhance these approaches by enabling the identification of individuals at risk of functional disability, with the added advantage of capturing phase-specific, time-sensitive movement patterns. The PCA analyzed the three-dimensional ROM of the lower limb joints and trunk, the angular velocity ranges, and the displacement and velocity ranges of CoM for each of the five TuG test phases. From an initial set of 150 variables, the PCMs filtered those with factor loadings above 0.8, retaining 59 variables. However, 21 of these variables exhibited low communalities and were excluded—a crucial step to ensure that only variables with meaningful contributions were retained, resulting in a KMO value above 0.5. The assessment of sample adequacy and data suitability for reduction is critical for PCA, yet it remains under reported in biomechanics and related fields [51].

A PCM of the TuG task was computed for the 38 retained variables, yielding 11 PCs that collectively explained 84.33% of the total variance. The phases contributing most to variance were the turn, walk-back, and walk-forward phases. Prior research has reported a significant decrease in step length, an increase in step time, and a reduction in step speed during the turn phase [14]. These variations may be reflected in the variance in the AP CoM velocity range in the turn phase, which was loaded onto the first PC. Furthermore, a correlation has been established between walking phases and the contraction and delay time of the vastus medialis muscle [52], which, in this study, is highlighted by the sagittal knee velocity range also loading onto PC1. Additionally, increased biceps femoris muscle stiffness has shown significant correlations with walk-forward and walk-back times [52]. This relationship may be represented in the PCM, where the sagittal hip ROM in the walk-forward and walk-back phases, along with the velocity range in the latter phase, is loaded onto PC1. Transverse hip ROM and velocity range in the walk-forward and walk-back phases were loaded onto PC10 and PC11, respectively. Furthermore, PC9 included ankle ROM and velocity range during the walk-forward phase in the transverse plane. An increased ankle plantar–flexor moment has been reported in elderly fallers as a movement strategy to enhance stability during sit-to-walk transitions [53]. However, research on the role of ankle kinematics in the transverse plane during this transition remains limited. Although the most commonly validated kinematic measures have focused on the sagittal plane, recent research has demonstrated that inertial measurement units show moderate-to-good agreement with optical motion capture systems for assessing kinematics across all three planes of motion: sagittal, transverse, and frontal [54]. Therefore, it is essential to incorporate these multidimensional metrics not only into gait analysis but also into transitional movements within the TUG test, such as turning and walking before and after a turn, which are highly representative of daily mobility in older adults.

PC2 included the frontal and sagittal trunk velocity ranges and frontal trunk ROM during the turn phase. This substantial contribution to variance may be attributed to the turning strategies adopted by older adults, such as smaller body inclination angles, longer trajectory lengths, and larger turning radii compared to younger adults [11]. The findings from PC8, which included mediolateral CoM displacement during the turn phase, further support these observations.

The third component was associated with sagittal knee and hip velocity range during the sit-to-walk phase. Sit-to-walk is a critical activity of daily living as gait initiation occurs immediately after standing, making this transition functionally significant [16]. As previously noted, older adults adopt a compensatory and conservative strategy, reaching an upright position with a brief pause before initiating gait [16]. The variance captured in this component may reflect differences in this strategy, as well as the maintenance of a neuromuscular reserve threshold to cope with movement uncertainties, which enables compensation before encountering physical limitations [12]. Prior research has also reported significantly reduced mean hip flexion ROM in elderly men compared to younger adults [12]. Furthermore, studies on the sit-to-stand task, which has been more extensively explored in the literature than the sit-to-walk task [16], have documented higher peak electromyography amplitudes of the tibialis anterior, vastus medialis, rectus femoris, biceps femoris, adductor longus, gluteus maximus, gluteus medius, and erector spine in older adults compared to young adults [55]. Additionally, older adults exhibit higher trunk flexion angular velocity during the sit-to-stand transition [55], which may explain the variance identified in PC6, encompassing tri-dimensional trunk velocity range. These findings reinforces the necessity of exploring the frontal and transverse planes, despite both sit-to-walk and sit-to-stand tasks being bipodal movements primarily characterized by kinematic changes in the sagittal plane [55,56].

The loading of tri-dimensional trunk velocity ranges was also observed during the walk-back phase (PC4), underscoring the significant role of trunk kinematics in older adults’ performance. A recent study suggests that gait-related differences in trunk kinematics emerge as early as 60 years of age, preceding the commonly used gait indicator of walking speed, which typically declines at around 80 years, and decreased trunk rotations may thus represent an early sign of age-related decline [57]. Furthermore, older adults exhibit reduced trunk rotations, smaller movements in the frontal plane, and a more flexed trunk posture during walking [57].

PC5 included transverse knee ROM and velocity range, along with frontal knee ROM during the turn phase. This finding aligns with previous research indicating that pivoting during walking involves greater internal–external rotation than straight-line gait [58].

PC7 included vertical CoM displacement during the turn-to-sit phase, which may reflect variance in older adults’ knee and lateral lumbar flexion during this phase, both of which tend to decrease with age [17,59].

The analysis of the differences between older adults with and without functional disability revealed variations in PC1 and PC4 scores. The variance captured by PC1 meaningfully distinguished older adults with and without functional disability, supporting its potential clinical relevance. In contrast, the remaining components (PC2–PC11) showed only small effect sizes, and most of their confidence intervals crossed zero, suggesting that group differences in these dimensions were minor or inconsistent. Taken together, these findings highlight PC1 as the most sensitive dimension for differentiating functional status, while other components appear to capture variability not strongly associated with disability. As previously noted, PC1 encompasses a set of variables from the walk-forward, turn, and walk-back phases, primarily related to sagittal hip kinematics. Although comparative kinematic analysis between older adults with and without functional disability are limited, it can be argued that differences in PC scores may be associated with variations in the ability of lower limb muscles to contract in a time-synchronized manner [52]. Additionally, hip abduction strength has been identified as a predictor of disability [60], which may further explain this differences. Additionally, the patterns identified suggest a hip-dominant (proximal) strategy and heightened CoM modulation when stability demands rise (walking backward and turning). Such reliance on proximal joints is consistent with age-related gait adaptations, including reduced hip extension/ROM and altered inter-segmental coordination, which prompt older adults to compensate via greater hip motion and velocity adjustments to maintain forward progression and balance [61,62].

Another distinguishing component between older adults with and without functional disability is the tri-dimensional trunk velocity range during the walk-back phase (PC4). The present study sample has a mean age of 67.86 years. As previously noted, differences in trunk kinematics emerge as early as 60 years of age, with decreased trunk rotations potentially serving as an early indicator of age-related decline [57] and, according to the present results, also for functional decline. Older adults typically exhibit reduced trunk rotations and smaller frontal plane motions, which can also be interpreted as a strategy to limit CoM excursion and enhance stability. Moreover, turning places additional demands on the trunk and CoM control and is a context where impaired trunk kinematics have been associated with instability and falls, underscoring the clinical relevance of trunk-centric components [57].

Despite the innovative insights presented, the results of this study should be interpreted in light of its limitations. The exclusion of individuals who were unable to perform the TuG test without upper limb assistance restricted the profile of older adults with functional disability. However, in the present study, participants were required to complete the task independently and without additional movement compensation to ensure the accurate computation of a TuG PCM that characterized older adults’ performance globally. This methodological decision likely excluded participants with more severe functional impairments, who may rely on distinct or more pronounced compensatory strategies. Therefore, the findings of this study may be most applicable to older adults with mild-to-moderate disability, and future research should explore whether similar biomechanical patterns are observed in individuals with more advanced functional decline. Additionally, although we carefully detailed our segmentation procedures and integrated different approaches to address the lack of consensus in the literature, it is important to recognize that methodological variability in TUG segmentation can affect the comparability and generalizability of results across studies. Different threshold criteria (e.g., based on GRF, joint kinematics, or CoM trajectories) may lead to subtle differences in the timing and characterization of the phases, which, in turn, could influence the weighting of variables in PCA or the interpretation of compensatory strategies. While our approach aimed to balance robustness and reproducibility, further efforts toward methodological standardization in TUG segmentation are needed to enhance cross-study comparisons and clinical translation.

This study introduces a novel approach to TuG assessment by evaluating not only temporal parameters but also the kinematics of the lower limbs and trunk segments. With recent advancements validating wearable gait analysis systems for measuring lower limb kinematics during the TUG test [63], identifying the most relevant kinematic metrics that distinguish functional status in older adults is crucial for effective and targeted monitoring. This method enabled the identification of key kinematic variables in each phase and provided a comprehensive description of older adults’ movement profiles during the task, as well as the kinematic differences between older adults with and without disability. Future research may incorporate the identified metrics into wearable monitoring systems.

## 5. Conclusions

The present study provides a novel approach to assessing the TuG test by combining temporal parameters analysis with PCA of lower limb, trunk, and CoM kinematics. Older adults with functional disability required significantly more time to complete the TuG test, particularly during the sit-to-walk, walk-forward, and walk-back phases. A PCM of the TuG test was computed for 38 retained variables from the phase-specific PCMs, yielding 11 PCs that collectively explained 84.33% of the total variance. The increased reliance on compensatory strategies, particularly during the sit-to-walk and turn phases, suggests a prioritization of stability at the cost of efficiency. These findings highlight key differences in movement patterns between older adults with and without functional disability, particularly in sagittal hip kinematics and tri-dimensional trunk velocity. Furthermore, the results emphasize the role of trunk kinematics as a potential early indicator of functional disability.

## Figures and Tables

**Table 1:** Demographic and clinical characterization of the sample. Data are represented by the mean and standard deviation for ordinal variables and by frequencies for nominal variables. The *p*-value reflects the comparison between older adults without disability (ND) and older adults with disability (D) by ^(a)^ Mann–Whitney U test, ^(b)^ chi-square test, and ^(c)^ independent samples *t*-test (* represent significant statistical differences).

		OA (n = 60)	ND (n = 35)	D (n = 25)	*p*-Value (Test Value)
**Demographic and Clinical Data**					
Age (years)		67.86 ± 6.46	66.34 ± 5.60	68.60 ± 6.77	0.147 (534) ^(a)^
Gender (n female/%)		38 (63.3)	19 (54.29)	19 (76)	0.085 (2.961) ^(b)^
BMI (kg/m^2^)		25.39 ± 2.96	25.22 ± 3.08	26.02 ± 2.66	0.298 (−1.049) ^(c)^
History of fall (n fallers/%)		22 (36.7)	11 (31.4)	11 (44)	0.469 (0.525) ^(b)^
Polypharmacy (n polymedicated/%)		13 (21.7)	2 (5.71)	11 (44)	<0.001 * (12.595) ^(b)^
Cognitive function (MMSE score)		28.74 ± 1.38	28.94 ± 1.31	28.68 ± 1.49	0.495 (394) ^(a)^
Self-reported physical activity (IPAQ MET-min/week)		3193.70 ± 2829.86	3186.46 ± 2964.91	3519.66 ± 2822.11	0.509 (393.5) ^(a)^
**Disability Indicators**					
Self-reported health	poor	29 (48.3)	8 (22.86)	21 (84)	<0.001 * (21.832) ^(b)^
	good	31 (51.6)	27 (77.14)	4 (16)	
Hand grip strength (kg)		27.39 ± 8.56	36.59 ± 39.86	25.07 ± 7.54	0.018 * (279.5) ^(a)^
One-leg standing time (s)		30.32 ± 22.77	38.83 ± 20.93	18.19 ± 20.72	<0.001 * (192.5) ^(a)^
ADL independence (Barthel Index score)		19.86 ± 0.35	19.97 ± 0.17	19.76 ± 0.44	0.013 (345) ^(a)^
IADL independence (Lawton and Brody score)		22.70 ± 1.23	23 ± 0.00	21.96 ± 2.67	0.002 (332.5) ^(a)^

**Table 2:** Mean and standard deviation of overall TuG completion time and respective phases of the total sample (OA) and older adults without (ND) and with (D) disability. Differences between ND and D groups analyzed by ^(a)^ Mann–Whitney U test or by ^(b)^ independent sample *t* test are represented by *p*-value (test value) (* represent significant statistical differences).

Time	OA (n = 60)	ND (n = 35)	D (n = 25)	*p*-Value (Test Value)
TuG	10.83 ± 2.02	10.34 ± 2.14	11.51 ± 1.63	0.006 * (254.00) ^(a)^
Sit-to-walk	1.36 ± 0.25	1.30 ± 0.27	1.44 ± 0.20	0.015 * (−2.21) ^(b)^
Walk-forward	2.21 ± 0.48	2.08 ± 0.49	2.39 ± 0.41	0.003 * (237.00) ^(a)^
Turn	1.78 ± 0.32	1.71 ± 0.29	1.87 ± 0.35	0.086 (323.00) ^(a)^
Walk-back	2.73 ± 0.71	2.56 ± 0.77	2.97 ± 0.55	0.007 * (259.00) ^(a)^
Turn-to-sit	2.72 ± 0.70	2.64 ± 0.76	2.84 ± 0.59	0.06 (312.00) ^(a)^

**Table 3:** Principal component model of the Timed Up and Go task. Parameters with loadings above 0.8 are in bold for each principal component (CoM—center of mass; ROM—range of motion).

Principal Component Model of Timed Up and Go Task											
Principal Component	1	2	3	4	5	6	7	8	9	10	11
Explained Variance (%)	22.21	11.31	9.18	7.34	6.57	5.86	5.45	4.98	4.31	3.70	3.42
Parameters	Loadings										
Sagittal hip ROM—Walk back	**0.877**	0.062	0.015	0.036	0.079	−0.036	−0.215	−0.018	−0.054	0.161	−0.041
Sagittal hip velocity range—Walk back	**0.865**	0.049	−0.113	0.179	0.154	−0.03	−0.259	0.118	0.114	0.089	0.091
Vertical CoM velocity range—Walk-back	**0.864**	0.043	0.094	0.056	0.056	−0.067	0.174	0.057	0.189	−0.079	0.066
Sagittal hip ROM—Walk forward	**0.855**	0.034	−0.266	−0.017	0.045	−0.004	0.008	0.117	−0.041	0.08	0.057
Sagittal knee velocity range—Walk forward	**0.826**	−0.007	−0.159	0.074	0.116	0.066	0.055	0.134	0.133	0.004	0.035
AP CoM velocity range—Turn	**0.816**	0.027	−0.175	−0.039	0.04	−0.153	0.188	0.198	0.154	0.094	0.11
Vertical CoM velocity range—Turn	**0.812**	0.05	−0.099	0.052	0.006	−0.112	0.224	0.046	0.186	−0.156	0.085
Frontal trunk velocity range—Turn	−0.063	**0.950**	0.022	0.043	−0.056	0.081	−0.023	−0.013	−0.008	0.01	0.02
Sagittal trunk velocity range—Turn	−0.08	**0.933**	−0.036	0.099	0.075	0.177	−0.056	−0.037	0.006	0.025	0.074
Frontal trunk ROM—Turn	0.157	**0.833**	0.063	−0.013	−0.121	0.034	0.182	0.017	−0.096	−0.021	−0.015
Sagittal knee velocity range—Sit-to-walk	−0.075	0.019	**0.892**	0.078	−0.017	0.018	−0.04	−0.045	0.094	0.139	0.016
Sagittal hip ROM—Sit-to-walk	−0.218	−0.026	**0.852**	0.065	0.164	0.144	0.068	−0.018	−0.033	−0.127	0.03
Sagittal trunk velocity range—Walk back	−0.094	0.075	0.151	**0.917**	0.077	−0.048	−0.043	−0.118	−0.063	−0.131	−0.031
Frontal trunk velocity range—Walk back	0.014	0.067	0.161	**0.904**	−0.021	−0.08	0.057	0.095	−0.076	−0.039	0.032
Transverse trunk velocity range—Walk back	0.307	−0.038	−0.038	**0.816**	0.044	−0.162	−0.032	−0.076	0.087	0.021	−0.089
Transverse knee velocity range—Turn	0.188	−0.014	−0.008	0.059	**0.904**	−0.05	0.013	0.132	−0.044	−0.03	0.115
Transverse knee ROM—Turn	0.102	−0.074	0.132	0.049	**0.853**	−0.079	−0.044	0.004	−0.093	0.122	−0.147
Frontal knee ROM—Turn	0.045	0.195	−0.047	0.041	**0.819**	−0.166	−0.098	0.014	0.08	0.162	0.016
Sagittal trunk velocity range—Sit-to-walk	−0.102	0.047	0.05	−0.101	−0.113	**0.873**	0.116	−0.019	0.084	0.02	0.113
Frontal trunk velocity range—Sit-to-walk	0.023	0.165	0.06	−0.042	−0.111	**0.846**	−0.167	0.043	−0.073	0.04	−0.068
Transverse trunk velocity range—Sit-to-walk	−0.131	0.211	0.017	−0.056	−0.048	**0.814**	−0.056	0.077	−0.002	−0.124	0.115
Vertical CoM displacement—Turn-to-sit	−0.038	0.091	0.154	0.038	−0.09	−0.12	**0.878**	0.015	−0.135	−0.083	0.053
ML CoM displacement—Turn	0.216	0.068	−0.035	−0.105	0.086	0.03	−0.024	**0.932**	0.103	−0.024	−0.008
ML CoM displacement—Walk back	0.146	−0.119	−0.092	0.047	0.097	0.04	0.068	**0.899**	0.013	−0.059	−0.054
Transverse ankle velocity range—Walk forward	0.215	−0.101	0.001	−0.009	0.087	−0.023	0.007	−0.002	**0.918**	0.085	−0.02
Transverse ankle ROM—Walk forward	0.255	0.014	−0.005	0.002	−0.181	0.032	−0.073	0.150	**0.883**	0.036	−0.005
Transverse hip ROM—Walk forward	−0.012	0.048	0.021	−0.004	0.071	0.015	0.001	−0.067	−0.03	**0.911**	0.076
Transverse hip velocity range—Walk forward	0.137	−0.04	−0.01	−0.074	0.16	−0.077	0.027	0.032	0.16	**0.887**	0.071
Transverse hip ROM—Walk back	0.077	0.054	0.003	0.008	−0.129	0.129	−0.058	−0.065	−0.086	0.076	**0.882**
Transverse hip velocity range—Walk back	0.230	0.061	0.048	−0.113	0.131	0.012	0.100	0.013	0.064	0.073	**0.875**

**Table 4:** Comparisons between principal component scores of older adults with and without disability according to the Mann–Whitney U test and respective effect size (* represent significant statistical differences).

Principal Component	*p*-Value (Test Value)	Cohen’s d	95% CI [Low, High]
1	0.035 * (278)	0.59	[0.06, 1.12]
2	0.094 (306)	0.34	[−0.18, 0.86]
3	0.146 (320)	0.32	[−0.85, 0.20]
4	0.035 * (278)	0.08	[−0.44, 0.60]
5	0.832 (399)	0.07	[−0.45, 0.59]
6	0.282 (344)	−0.04	[−0.55, 0.48]
7	0.061 (293)	0.44	[−0.10, 0.98]
8	0.206 (332)	−0.32	[−0.85, 0.21]
9	0.179 (327)	0.37	[−0.17, 0.91]
10	0.783 (395)	0.06	[−0.47, 0.59]
11	0.906 (405)	0.12	[−0.41, 0.66]

## Data Availability

Data is unavailable due to privacy.

## Supplementary Materials

The following supporting information can be downloaded at https://www.mdpi.com/article/10.3390/bios15090621/s1: Table S1: Principal component model of tridimensional trunk, hip, knee, and ankle range of motion (ROM), joint velocity range, and center of mass (CoM) displacement and velocity range in the anteroposterior (AP), mediolateral (ML), and vertical directions during the sit-to-walk phase of the TuG task. Kaiser–Meyer–Olkin value of 0.556 and Bartlett’s test of sphericity < 0.05. Parameters with loadings > 0.800 are in bold. Table S2: Principal component model of tridimensional trunk, hip, knee, and ankle range of motion (ROM), joint velocity range, and center of mass (CoM) displacement and velocity range in the anteroposterior (AP), mediolateral (ML), and vertical directions during the walking-forward phase of the TuG task. Kaiser–Meyer–Olkin value of 0.635 and Bartlett’s test of sphericity < 0.05. Parameters with loadings > 0.800 are in bold. Table S3: Principal component model of tridimensional trunk, hip, knee, and ankle range of motion (ROM), joint velocity range, and center of mass (CoM) displacement and velocity range in the anteroposterior (AP), mediolateral (ML), and vertical directions during the turn phase of the TuG task. Kaiser–Meyer–Olkin value of 0.754 and Bartlett’s test of sphericity < 0.05. Parameters with loadings > 0.800 are in bold. Table S4: Principal component model of tridimensional trunk, hip, knee, and ankle range of motion (ROM), joint velocity range, and center of mass (CoM) displacement and velocity range in the anteroposterior (AP), mediolateral (ML), and vertical directions during the walking-back phase of the TuG task. Kaiser–Meyer–Olkin value of 0.653 and Bartlett’s test of sphericity < 0.05. Parameters with loadings > 0.800 are in bold. Table S5: Principal component model of tridimensional trunk, hip, knee, and ankle range of motion (ROM), joint velocity range, and center of mass (CoM) displacement and velocity range in the anteroposterior (AP), mediolateral (ML), and vertical directions during the turn-to-sit phase of the TuG task. Kaiser–Meyer–Olkin value of 0.594 and Bartlett’s test of sphericity < 0.05. Parameters with loadings > 0.800 are in bold.

## Abbreviations

The following abbreviations are used in this manuscript:

ADL	Activities of Daily Living
CoM	Center of Mass
GRF	Ground Reaction Forces
IADL	Instrumental Activities of Daily Living
IPAQ	International Physical Activity Questionnaire
KMO	Kaiser–Meyer–Olkin
MMSE	Mini Mental State Examination
OLST	One-Leg Standing Test
PC	Principal Components
PCA	Principal Component Analysis
PCM	Principal Component Model
ROM	Range of Motion
SD	Standard Deviation
SRH	Self-Reported Health
STROBE	Strengthening the Reporting of Observational Studies in Epidemiology
iTuG	Instrumented Timed Up and Go
TuG	Timed Up and Go
