# Supplementary material for: Kinematic Biomarkers of Functional Disability in Older Adults: Analysis of the Timed Up and Go Test
Source: Biosensors (Basel). 2025 Sep 19;15(9):621. doi: 10.3390/bios15090621 (PMC12467501; doi:10.3390/bios15090621)
Supplement: Supplementary file 1 [file biosensors-15-00621-s001.zip › biosensors-3819568-supplementary.pdf]

biosensors

MDPI

Supplementary material

Kinematic Biomarkers of Functional Disability in Older Adults: Analysis of the Timed Up and Go Test

Juliana Moreira [1,2], Bruno Cunha [3], José Félix [1,4,5], Rubim Santos [1] and Andreia S. P. Sousa [1,*]

[1] CIR, E2S, Polytechnic of Porto, Rua Dr. António Bernardino de Almeida, 4249-015 Porto, Portugal;
[2] Research Center in Physical Activity, Health and Leisure, Faculty of Sports, University of Porto, 4200-450 Porto, Portugal
[3] CINTESIS@RISE, CINTESIS.UPT, Portucalense University, Rua Dr. António Bernardino de Almeida 541, 4200-072 Porto, Portugal;
[4] Department of Physiotherapy, Institute of Health of the North—Escola Superior de Saúde do Vale do Ave (ESSVA), Cooperativa de Ensino Superior Politécnico e Universitário (CESPU), 4760-409 Vila Nova de Famalicão, Portugal
[5] Department of Medical Sciences, University of Aveiro, Agras do Crasto, Campus Universitário de Santiago, 3810-193 Aveiro, Portugal
* Correspondence: asp@ess.ipp.pt (A.S.P.S)

Table S1. Principal component model of tridimensional trunk, hip, knee and ankle range of motion (ROM), joint velocity range, and center of mass (CoM) displacement and velocity range in antero-posterior (AP), mediolateral (ML) and vertical directions during the Sit-to-walk phase of TuG task. Kaiser-Meyer-Olkin value of 0.556 and Bartlett's Test of Sphericity <0.05. Parameters with loadings >0.800 are in bold.

**Sit-to-walk phase**								
Principal component	1	2	3	4	5	6	7	8
Explained variance (%)	20.99	16.84	11.88	9.13	5.96	4.36	3.92	3.35
**Sagittal knee ROM**	**0.890**	0.02	-0.216	-0.107	-0.111	0.184	0.164	-0.167
**Sagittal hip ROM**	**0.825**	0.046	0.129	-0.175	0.128	0.079	0.136	0.150
**Sagittal knee velocity range**	**0.823**	-0.018	0.033	0.014	0.15	-0.051	0.237	-0.048
Vertical CoM displacement	0.780	0.074	0.374	-0.106	-0.262	0.164	-0.003	-0.122
Sagittal trunk ROM	0.676	0.121	-0.085	0.154	-0.029	-0.095	-0.353	0.310
Frontal knee ROM	0.671	0.052	-0.404	0.117	0.238	0.092	0.013	0.120
**Transverse trunk velocity range**	0.004	**0.865**	-0.043	-0.117	0.041	0.035	0.090	0.033
**Frontal trunk joint velocity range**	-0.020	**0.852**	-0.014	-0.132	0.173	0.118	0.073	0.045
**Sagittal trunk joint velocity range**	0.145	**0.848**	0.146	-0.009	-0.044	0.094	-0.217	-0.057
Transverse trunk ROM	0.014	0.720	0.052	0.03	-0.178	0.250	0.102	0.383
Transverse hip velocity range	0.354	0.509	0.063	0.01	0.287	0.024	0.327	0.347
Transverse hip ROM	0.436	0.476	-0.147	0.153	0.309	0.040	0.155	0.294
**Vertical CoM velocity range**	0.110	-0.045	**0.846**	0.048	-0.193	-0.118	0.120	-0.043
AP CoM displacement	-0.045	0.024	0.787	0.219	0.250	0.162	-0.241	-0.076
Sagittal hip velocity range	0.087	0.178	0.785	0.102	0.150	-0.081	0.189	0.389
AP CoM velocity range	-0.454	-0.029	0.639	0.336	0.208	-0.146	-0.01	-0.089
Sagittal ankle velocity range	-0.273	-0.05	0.553	0.513	0.046	-0.125	0.154	0.044
Frontal ankle ROM	0.150	0.221	0.504	0.403	-0.420	0.138	-0.065	0.014
Transverse ankle ROM	0.216	-0.091	-0.019	0.764	0.037	0.141	0.298	0.078
Sagittal ankle ROM	-0.241	-0.194	0.139	0.755	0.153	-0.039	-0.124	0.133
Transverse ankle velocity range	0.079	0.047	0.158	0.752	0.156	-0.283	0.232	0.025
Frontal ankle velocity range	-0.155	0.001	0.368	0.674	-0.117	-0.004	-0.053	-0.228

**Transverse knee velocity range**	0.129	0.012	0.091	0.153	**0.882**	-0.078	0.058	-0.083
Frontal Hip velocity range	-0.013	0.485	0.081	0.087	0.695	0.045	0.179	0.153
**ML CoM displacement**	0.159	0.044	-0.075	-0.149	-0.064	**0.859**	-0.005	0.151
ML CoM velocity range	0.076	0.394	-0.106	-0.006	0.078	0.78	-0.083	-0.176
Frontal hip ROM	0.056	0.267	0.189	0.115	-0.427	0.54	0.291	0.262
Frontal knee velocity range	0.193	0.02	0.031	0.245	0.191	-0.056	0.737	0.079
Transverse knee ROM	0.39	0.248	0.057	0.09	-0.104	0.092	0.435	-0.222
Frontal Trunk ROM	0.013	0.511	0.048	0.028	-0.069	0.121	-0.044	0.68

Table S2. Principal component model of tridimensional trunk, hip, knee and ankle range of motion (ROM), joint velocity range, and center of mass (CoM) displacement and velocity range in antero-posterior (AP), mediolateral (ML) and vertical directions during the Walking forward phase of TuG task. Kaiser-Meyer-Olkin value of 0.635 and Bartlett's Test of Sphericity <0.05. Parameters with loadings >0.800 are in bold.

**Walking forward phase**									
Principal component	1	2	3	4	5	6	7	8	9
Explained variance (%)	28.22	12.90	9.37	7.54	6.87	5.57	4.63	3.58	3.40
**Sagittal knee velocity range**	**0.874**	0.210	-0.004	0.144	0.19	-0.06	-0.03	0.032	-0.044
**Sagittal hip ROM**	**0.865**	0.037	0.025	0.144	0.11	-0.012	0.086	0.067	0.221
**Vertical CoM velocity range**	**0.800**	0.106	0.02	0.04	0.005	0.09	0.096	0.394	0.123
Sagittal Hip velocity range	0.777	0.307	0.036	0.267	0.132	0.04	0.194	-0.099	0.243
AP CoM velocity range	0.728	0.136	0.075	0.072	-0.114	0.034	0.038	0.141	0.015
Sagittal knee ROM	0.724	0.07	0.185	0.044	0.200	-0.002	-0.314	-0.027	-0.311
Vertical CoM displacement	0.648	-0.04	-0.024	-0.095	-0.207	-0.008	0.153	0.644	0.051
Transverse trunk ROM	0.549	0.258	0.072	0.424	0.208	-0.118	-0.009	-0.008	0.310
Frontal hip velocity range	0.485	-0.013	0.39	0.312	0.44	0.205	0.089	-0.197	-0.054
**Sagittal ankle ROM**	0.17	**0.865**	-0.1	-0.026	0.098	0.096	0.015	-0.116	-0.042
**Transverse ankle velocity range**	0.11	**0.85**	0.172	0.165	-0.038	-0.135	0.071	0.142	0.098
**Transverse ankle ROM**	0.057	**0.835**	0.019	0.151	0.157	-0.109	0.192	0.163	0.118
Sagittal ankle velocity range	0.442	0.755	0.047	0.075	0.052	0.106	0.104	-0.174	-0.058
**Transverse hip velocity range**	0.117	0.191	**0.884**	0.069	0.046	0.135	0.103	0.076	0.083
**Transverse hip ROM**	-0.04	-0.063	**0.86**	0.104	0.064	0.132	0.083	0.017	0.059
**Transverse knee velocity range**	0.163	0.047	**0.837**	0	-0.089	0.189	0.067	0.007	-0.035
**Sagittal trunk velocity range**	0.08	0.1	0.045	**0.907**	-0.044	0.15	-0.041	0.106	-0.172
**Frontal trunk velocity range**	0.117	0.032	0.107	**0.889**	0.147	0.01	-0.02	-0.02	-0.028
Transverse trunk velocity range	0.352	0.186	-0.008	0.788	-0.066	0.103	0.186	-0.014	0.019
**ML CoM displacement**	-0.071	0.057	-0.052	-0.054	**0.892**	0.127	0.17	0.015	-0.081
**ML CoM velocity range**	0.24	0.114	0.008	0.055	**0.847**	0.034	0.106	0.11	0.152
Frontal hip ROM	0.401	0.292	0.102	0.279	0.46	0.143	-0.084	-0.197	0.336
**Frontal knee velocity range**	0.073	0.045	0.257	0.196	0.223	**0.856**	-0.003	-0.107	-0.066
Frontal knee ROM	0.004	-0.039	0.434	0.18	-0.016	0.721	0.004	0.056	0.253
Transverse knee ROM	-0.063	-0.125	0.604	-0.109	0.038	0.637	-0.015	-0.014	0.098
**Frontal ankle velocity range**	0.163	0.195	0.137	-0.019	0.024	0.106	**0.868**	0.006	-0.062
**Frontal ankle ROM**	-0.036	0.111	0.136	0.103	0.315	-0.133	**0.82**	-0.097	-0.131
**Sagittal trunk ROM**	0.186	0.033	0.089	0.087	0.152	-0.063	-0.137	**0.897**	0.013
AP CoM displacement	-0.14	-0.059	-0.087	0.22	-0.035	-0.164	0.161	-0.032	-0.797
Frontal trunk ROM	0.373	0.091	0.268	0.097	0.398	-0.288	-0.077	0.126	0.431

Table S3. Principal component model of tridimensional trunk, hip, knee and ankle range of motion (ROM), joint velocity range, and center of mass (CoM) displacement and velocity range in

anteroposterior (AP), mediolateral (ML) and vertical directions during the Turn phase of TuG task. Kaiser-Meyer-Olkin value of 0.754 and Bartlett's Test of Sphericity <0.05. Parameters with loadings >0.800 are in bold.

**Turn phase**							
Principal component	1	2	3	4	5	6	7
Explained variance (%)	35.41	15.93	10.52	6.58	5.21	4.70	3.46
**Vertical CoM velocity range**	**0.903**	0.033	-0.012	0.128	0.142	0	-0.055
**Vertical CoM displacement**	**0.889**	0.029	-0.082	0.062	-0.028	-0.123	0.108
**AP CoM velocity range**	**0.849**	-0.006	0.072	0.263	0.117	0.252	0.111
Sagittal knee velocity range	0.753	0.031	0.192	0.138	0.389	0.327	0.062
Sagittal knee ROM	0.715	0.044	0.303	0.11	0.269	0.315	0.017
Sagittal hip ROM	0.682	0.241	0.14	0.181	0.176	0.321	0.284
Sagittal hip velocity range	0.644	0.492	0.212	0.216	0.3	0.207	0.076
**Frontal trunk velocity range**	-0.06	**0.951**	-0.085	-0.03	0.101	-0.065	-0.035
**Sagittal trunk velocity range**	-0.096	**0.95**	0.026	-0.017	0.082	-0.022	-0.065
**Frontal trunk ROM**	0.162	**0.834**	-0.12	0.024	-0.117	0.161	0.266
**Frontal hip velocity range**	0.159	**0.801**	0.357	-0.03	0.208	0.066	-0.149
Sagittal trunk ROM	0.146	0.767	0.218	-0.04	-0.032	0.203	-0.09
Transverse trunk velocity range	-0.026	0.648	0.043	0.232	-0.31	-0.16	0.267
Frontal hip ROM	0.406	0.602	0.29	0.222	-0.006	0.187	0.25
**Transverse knee velocity range**	0.175	-0.002	**0.897**	-0.012	0.136	0.114	-0.009
**Transverse knee ROM**	-0.02	-0.07	**0.855**	0.012	0.053	-0.011	0.243
**Frontal knee ROM**	-0.036	0.172	**0.843**	0.168	0.097	-0.006	0.091
Transverse hip velocity range	0.225	0.261	0.754	0.027	-0.119	0.08	-0.342
Transverse ankle ROM	0.075	0.067	0.075	0.777	0.174	0.361	-0.046
Sagittal ankle ROM	0.474	-0.001	-0.058	0.756	-0.05	-0.043	0.268
Sagittal ankle velocity range	0.534	0.037	0.057	0.747	0.144	0.152	0.102
Transverse ankle velocity range	0.155	-0.032	0.342	0.62	0.484	0.314	-0.065
**ML CoM displacement**	0.188	0.006	0.032	0.126	**0.855**	0.165	0.202
ML CoM velocity range	0.531	0.012	0.049	0.135	0.734	0.164	0.064
Frontal knee velocity range	0.407	0.139	0.406	0.235	0.453	0.058	-0.051
**Frontal ankle ROM**	0.191	0.047	0.118	0.206	0.105	**0.846**	0.127
Frontal ankle velocity range	0.261	0.166	-0.057	0.245	0.293	0.774	-0.169
AP CoM displacement	0.36	0.22	0.119	0.068	0.198	-0.059	0.677
Transverse hip ROM	0.064	0.346	0.514	-0.128	-0.323	0.006	-0.557
Transverse trunk ROM	0.483	0.079	0.261	0.089	-0.069	0.305	0.486

Table S4. Principal component model of tridimensional trunk, hip, knee and ankle range of motion (ROM), joint velocity range, and center of mass (CoM) displacement and velocity range in antero-posterior (AP), mediolateral (ML) and vertical directions during the Walking back phase of TuG task. Kaiser-Meyer-Olkin value of 0.653 and Bartlett's Test of Sphericity <0.05. Parameters with loadings >0.800 are in bold.

**Walking back phase**								
Principal component	1	2	3	4	5	6	7	8
Explained variance (%)	28.48	11.35	9.43	7.29	6.51	5.37	4.87	4.10
**Sagittal hip ROM**	**0.890**	0.009	-0.004	0.09	0.037	-0.01	0.066	-0.122
**Sagittal knee velocity range**	**0.843**	0.101	0.043	0.076	0.147	0.081	0.079	0.069
**Sagittal hip velocity range**	**0.842**	0.202	0.122	0.192	0.188	0.062	0.213	-0.156
**Vertical CoM velocity range**	**0.813**	0.221	-0.015	0.157	-0.18	0.075	0.187	0.163
Sagittal knee ROM	0.778	0.042	0.07	-0.065	0.05	0.093	-0.082	0.193
Transverse trunk ROM	0.686	0.184	0.296	-0.117	0.067	-0.14	0.088	0.162
Frontal hip ROM	0.636	0.319	0.016	0.12	0.359	-0.226	-0.048	-0.015
Vertical CoM displacement	0.596	0.07	-0.069	0.158	-0.332	0.176	0.162	0.395
Frontal trunk ROM	0.536	0.154	0.147	-0.012	0.036	-0.365	0.306	0.45
**Transverse ankle velocity range**	0.100	**0.801**	0.069	0.137	0.336	-0.03	0.094	-0.018
Transverse ankle ROM	0.054	0.772	0.157	-0.135	0.22	0.149	-0.082	0.211
Sagittal ankle ROM	0.234	0.76	0.03	0.051	-0.287	0.169	0.105	-0.143
Sagittal ankle velocity range	0.523	0.72	0.004	0.116	0.004	0.039	0.143	-0.08
**Sagittal trunk velocity range**	-0.057	0.05	**0.938**	-0.048	-0.045	0.152	-0.052	-0.016
**Frontal trunk velocity range**	0.033	0.037	**0.928**	0.039	-0.015	0.078	0.07	0.049
**Transverse trunk velocity range**	0.333	0.157	**0.813**	-0.105	0.089	0.117	0	0.123
**Transverse hip velocity range**	0.103	0.095	-0.064	**0.916**	-0.05	-0.083	0.08	-0.106
**Transverse hip ROM**	-0.012	-0.128	-0.01	**0.826**	0.046	0.043	-0.161	0.188
Transverse knee velocity range	0.14	0.242	-0.008	0.656	0.373	-0.052	0.314	-0.158
Frontal hip velocity range	0.545	0.092	-0.039	0.592	0.277	0.09	-0.086	0.099
**Frontal knee ROM**	0.063	0.114	-0.032	0.062	**0.812**	-0.095	0.151	-0.017
Transverse knee ROM	0.087	0.068	0.019	0.091	0.764	0.187	0.274	-0.068
Frontal knee velocity range	0.346	0.158	0.048	0.272	0.527	0.29	-0.087	0.204
**AP CoM displacement**	-0.242	-0.042	0.156	-0.154	-0.011	**0.874**	0.03	-0.059
Frontal ankle velocity range	0.239	0.323	0.316	0.068	0.134	0.651	-0.147	0.143
Frontal ankle ROM	0.211	0.442	0.223	0.048	0.225	0.586	-0.025	0.076
AP CoM velocity range	0.389	0.1	-0.088	0.175	-0.386	0.47	0.215	-0.046
**ML CoM velocity range**	0.167	0.042	0.033	0.082	0.138	0.045	**0.857**	-0.069
**ML CoM displacement**	0.098	0.059	-0.02	-0.087	0.149	-0.064	**0.831**	0.138
**Sagittal trunk ROM**	0.132	-0.03	0.101	0.022	-0.009	0.033	0.016	**0.885**

Table S5. Principal component model of tridimensional trunk, hip, knee and ankle range of motion (ROM), joint velocity range, and center of mass (CoM) displacement and velocity range in antero-posterior (AP), mediolateral (ML) and vertical directions during the Turn-to-sit phase of TuG task. Kaiser-Meyer-Olkin value of 0.594 and Bartlett's Test of Sphericity <0.05. Parameters with loadings >0.800 are in bold.

**Turn-to-sit phase**									
Principal component	1	2	3	4	5	6	7	8	9
Explained variance (%)	20.96	14.22	9.16	8.02	7.04	5.60	4.20	3.90	3.71
**AP CoM velocity range**	**0.844**	0.166	0.027	0.199	-0.045	-0.229	-0.008	0.155	0.06
AP CoM displacement	0.784	0.345	0.074	0.064	0.041	-0.118	0.037	0.036	-0.086
Frontal Trunk ROM	0.703	-0.173	-0.039	-0.017	0.198	0.226	0.241	0.036	0.061
Frontal knee velocity range	0.604	-0.054	0.114	0.34	-0.079	0.338	-0.259	0.214	0.083
Sagittal hip velocity range	0.568	0.25	0.395	0.125	-0.113	-0.006	-0.033	0.26	0.409
Transverse trunk ROM	0.538	-0.371	0.204	0.26	0.14	-0.102	0.035	-0.115	0.447
**Vertical CoM displacement**	0.165	**0.890**	0.059	-0.077	0.186	-0.055	-0.068	-0.184	-0.002
Sagittal knee ROM	-0.248	0.755	0.128	-0.132	0.398	-0.005	-0.094	-0.175	-0.116
Transverse CoM velocity range	0.492	0.625	0.086	0.137	-0.026	-0.06	-0.054	0.032	0.362
Sagittal hip ROM	0.192	0.585	0.367	-0.329	0.13	-0.158	0.071	-0.107	-0.04
Frontal ankle ROM	0.414	0.5	-0.182	0.323	-0.305	0.091	0.368	0.188	-0.042
**Transverse hip velocity range**	0.006	0.237	**0.805**	-0.019	0.031	-0.02	-0.279	-0.009	0.23
Frontal hip velocity range	0.186	-0.175	0.694	0.129	-0.267	0.236	0.219	0.129	-0.139
Transverse hip ROM	0.063	0.133	0.675	-0.069	0.192	0.231	0.184	-0.299	-0.139
Sagittal knee velocity range	0.104	0.502	0.556	0.172	-0.097	-0.129	-0.039	0.051	-0.049
**Transverse ankle velocity range**	0.166	-0.22	0.143	**0.807**	-0.021	0.027	-0.047	0.161	0.081
Transverse ankle ROM	0.24	-0.056	-0.119	0.755	0.04	0.311	0.135	0.102	-0.081
Frontal ankle velocity range	0.166	0.401	0.196	0.588	-0.137	-0.04	0.063	0.312	-0.131
ML CoM displacement	-0.06	-0.004	-0.043	-0.046	0.788	0.212	0.029	0.079	-0.031
ML CoM velocity range	0.295	0.194	-0.018	-0.067	0.759	-0.15	0.053	0.128	-0.04
Sagittal trunk ROM	-0.046	0.382	-0.023	0.46	0.615	0.065	-0.101	-0.015	0.137
**Transverse knee ROM**	0.056	-0.037	-0.062	0.071	0.142	**0.892**	-0.031	-0.009	-0.075
Frontal knee ROM	-0.134	-0.087	0.275	0.13	0.005	0.65	0.092	0.083	0.14
Transverse knee velocity range	-0.017	-0.012	0.527	0.099	-0.114	0.549	-0.075	0.39	0.12
**Frontal trunk velocity range**	0.068	-0.214	0.105	-0.111	-0.012	0.01	**0.855**	0.056	0.061
Sagittal trunk velocity range	0.011	0.182	-0.1	0.239	0.057	-0.006	0.779	0.036	0.319
Sagittal ankle ROM	0.154	-0.169	-0.024	0.241	0.185	0.179	0.156	0.761	-0.058
Sagittal ankle velocity range	0.357	-0.057	0.078	0.413	0.175	0.04	0.061	0.731	0.045
Frontal hip ROM	0.438	0.146	0.274	0.266	0.113	0.055	0.185	-0.457	-0.018
Transverse trunk velocity range	0.093	-0.035	-0.046	-0.097	-0.031	0.098	0.344	-0.014	0.796
